# Effects of Titanium-Implanted Dose on the Tribological Properties of 316L Stainless Steel

**DOI:** 10.3390/ma14061482

**Published:** 2021-03-18

**Authors:** Wei Wang, Zhiqiang Fu, Lina Zhu, Wen Yue, Jiajie Kang, Dingshun She, Xiaoyong Ren, Chengbiao Wang

**Affiliations:** 1 School of Engineering and Technology, China University of Geosciences (Beijing), Beijing 100083, China; wangwei_2021_luck@163.com (W.W.); fuzhiqiang@cugb.edu.cn (Z.F.); yuewen@cugb.edu.cn (W.Y.); kangjiajie@cugb.edu.cn (J.K.); shedingshun@163.com (D.S.); 2 Zhengzhou Institute, China University of Geosciences (Beijing), Zhengzhou 451283, China; wangchengbiao@cugb.edu.cn; 3 School of Mechanical Electronic & Information Engineering, China University of Mining & Technology (Beijing), Beijing 100083, China; xiaoyong_ren@cumtb.edu.cn; 4 Zhengzhou Institute of Multipurpose Utilization of Mineral Resources, Chinese Academy of Geological Sciences, Zhengzhou 450006, China

**Keywords:** titanium ion implantation, dose, austenitic stainless steel, nano-hardness, tribological properties

## Abstract

The effects of titanium (Ti) ion-implanted doses on the chemical composition, surface roughness, mechanical properties, as well as tribological properties of 316L austenitic stainless steel are investigated in this paper. The Ti ion implantations were carried out at an energy of 40 kV and at 2 mA for different doses of 3.0 × 10^16^, 1.0 × 10^17^, 1.0 × 10^18^, and 1.7 × 10^18^ ions/cm^2^. The results showed that a new phase (Cr_2_Ti) was detected, and the concentrations of Ti and C increased obviously when the dose exceeded 1.0 × 10^17^ ions/cm^2^. The surface roughness can be significantly reduced after Ti ion implantation. The nano-hardness increased from 3.44 to 5.21 GPa at a Ti ion-implanted dose increase up to 1.0 × 10^18^ ions/cm^2^. The friction coefficient decreased from 0.78 for un-implanted samples to 0.68 for a sample at the dose of 1.7 × 10^18^ ions/cm^2^. The wear rate was slightly improved when the sample implanted Ti ion at a dose of 1.0 × 10^18^ ions/cm^2^. Adhesive wear and oxidation wear are the main wear mechanisms, and a slightly abrasive wear is observed during sliding. Oxidation wear was improved significantly as the implantation dose increased.

## 1. Introduction

Austenitic stainless steel has been widely applied in various industries, such as food processing, chemical engineering, and biomaterial applications, for its excellent resistance to corrosion [1,2,3]. However, the poor wear resistance limits its wider application and reduces its service life to some extent [4]. Many studies have been conducted to improve its mechanical properties and tribological properties [1,5,6,7]. The surface properties of materials have a very important influence on their friction and wear properties [8]. Ion implantation is an excellent technology for surface modification, which has been developed rapidly for its excellent advantages such as the implanted atoms being not restricted by thermodynamic equilibrium conditions and solid solubility, and there are no obvious interface between the modification layer and matrix [9,10,11,12].

Ion implantation not only could change the chemical composition and microstructure of the materials surface but also could help improve its tribological properties [13,14], corrosion resistance [15,16], and oxidation resistance [17]. It is reported that titanium (Ti) ion implantation could improve the wear resistance of several different steels through the formation of an amorphous surface layer [8,13]. At the same time, Ti ion implantation can also improve the corrosion resistance of austenitic stainless steel, which is the main advantage of austenitic stainless steel use [18]. Therefore, a lot of studies have been conducted on Ti ion implantation in austenitic stainless steels [8,10,18,19,20,21]. Evans et al. [8] investigated the wear properties of 316 stainless steel implanted with Ti for ion doses in the range (2.3–5.1) × 10^16^ ions/cm^2^ and found that implantation improved the hardness and decreased the friction. Youssef et al. [19] found that the surface hardness of stainless steel increased with Ti ion implantation dose increases, while no obvious improvement in the tribological properties was observed. The dose of Ti ion implantation has an important effect on improving the surface hardness and tribological properties of austenitic stainless steel. Previous works mainly focused on the surface modification of 316L stainless steel by titanium implantation carried out at intermediate energy with the dose increased up to 10^17^ ions/cm^2^. However, few researchers studied the effect of higher implantation doses on mechanical and tribological properties of 316L stainless steel.

It is reported that an amorphous layer would form on the surface as the implantation dose increases and that the thickness of the layer increases as the implantation dose increases [20]. Moreover, the formation of the amorphous layer could decrease the friction coefficient, which plays an important role in improvements in tribological properties of samples [21]. It is necessary to provide a further study on the effect of higher Ti ion-implanted dose on the tribological and mechanical properties of 316L stainless steel.

In this paper, the chemical composition, surface morphology, surface hardness, and wear properties of 316L stainless steel implanted with different doses of Ti ions are investigated. The doses range from 3.0 × 10^16^ to 1.7 × 10^18^ ions/cm^2^.

## 2. Materials and Methods

### 2.1. Sample Preparation

In this work, 316L stainless steel piece (50 mm × 30 mm × 3 mm) (TAIYUAN IRON & STEEL (GROUP) CO., LTD, Taiyuan, China) was chosen as the substrate for titanium (Ti) ion implantation. The chemical composition (wt.%) was C ≤ 0.03, Si ≤ 1.00, Mn ≤ 2.00, P ≤ 0.035, S ≤ 0.03, Ni 12.0–15.0, Cr 16.0–18.0, Mo 2.0–3.0, and Fe balance. In order to eliminate the influence of work hardening on the surface of stainless steel, the sample was subjected to vacuum annealing at 1050 ℃ for 2 h. The hardness of samples after annealing was 125 ± 3 HV_0.02_. Then, the surface of the samples was polished by electrochemical, and all the samples were ultrasonic cleaned sequentially with acetone, alcohol, and deionized (DI) water for 30 min. Ion implantation was carried out on ISB-700 type multifunctional coating device equipped with a metal vapor vacuum arc (MEVVA) ion source. Before Ti+ implantation, the mechanically polished and cleaned samples were further sputter cleaned in an argon atmosphere for 10 min. The Ti ion implantation was performed at an extraction voltage of 40 kV and an ion beam current of 2 mA. The temperature of the sample table was controlled below 100 ℃ during the ion implantation process. Four groups of Ti ion-implanted doses (3.0 × 10^16^, 1.0 × 10^17^, 1.0 × 10^18^, and 1.7 × 10^18^ ions/cm^2^) were prepared in this work.

### 2.2. Sample Characterization

The surface roughness was measured by a Nano-map profile-meter (NanoMap-D) (AEP Technology, Santa Clara, CA, USA), which was evaluated at an area size of 1018 × 1018 μm^2^. Eight groups of surface roughness were measured at different positions on the surface, and the average value was used to improve the statistical accuracy. The surface topography of samples before and after the wear test were examined by scanning electron microscope (SEM, JSM-6460LV) (JEOL, Tokyo, Japan) at a voltage of 20 kV. The depth profiles of the implanted layer were measured by Auger electron spectroscopy (AES, PHI-700 SAM) (ULVAC-PHI, Kanagawa, Japan). X-ray diffraction (XRD, D/max 2500) (Bruker, Billerica, MA, USA) at an incident angle of 2° was used to identify the phases of the implanted layers with Cu Ka radiation (λ = 0.15418 nm) through continuous scanning mode at a speed of 4°/min. Furthermore, the scan step size was 0.02°. Nano-hardness measurements of the modified layers were performed on a nano-indenter (MTS-XP) with a three-sided pyramidal diamond (Berkovich) indenter (Keysight technologies, Santa Rosa, CA, USA). A maximum indentation depth of 500 nm was adopted with the test method of continuous stifness method (CSM), and five indents were applied under each load for statistical purpose. According to the load-displacement curve, the elastic modulus was calculated from the slope of the unloading curve by the method of Oliver-Pharr.

### 2.3. Wear Test

Dry wear tests were performed in the rotation mode on a ball-on-disc tribometer (UMT-3) (CETR, Campbell, CA, USA). The counter-pair was Si_3_N_4_ ball with a diameter of 5 mm. The applied load, sliding velocity, and radius rotation were 1 N, 25 mm/s, and 4 mm, respectively. The sliding distance was 45 m. Three different positions on each sample were randomly selected to perform the friction and wear tests. The profiles of the wear tracks were obtained by means of a Nano-map profile-meter (NanoMap-D) (AEP technology, Santa Clara, CA, USA). Six groups of cross-sectional areas were measured at different positions on the track, and the average value was used to improve the statistical accuracy. The wear rate was calculated by the following formula:

*W_R_* = *W_v_*/(*PS*) (1)

where *W_v_* is the wear volume (m^3^), *P* is the applied load (N), and *S* is the sliding distance (m). The *W_v_* was evaluated by the following formula:

*W_v_* = 2*πRA* (2)

where *A* is the cross sectional area of the wear track (m^2^) and *R* is the length of the wear track (m). *S* was calculated by the following formula:

*S* = *vt* (3)

where *v* is sliding velocity (mm/s) and *t* is wear time (s).

## 3. Results

### 3.1. Chemical Composition

**Figure 1:**
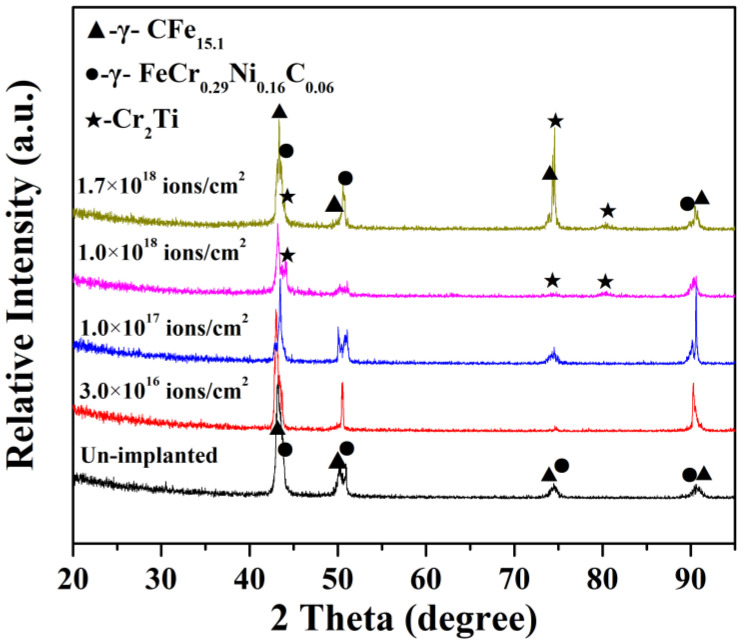
XRD patterns of 316L stainless steel with different Ti ion implantation doses.

Figure 1 shows the XRD patterns of the 316L stainless steel with different Ti ion implantation doses. The main observed XRD peaks of 316L stainless steel without Ti ion implantation correspond to two main phases (γ-CFe_15.1_ and γ-FeCr_0.29_Ni_0.16_C_0.06_). When the dose of Ti ion implantation was more than 1.0 × 10^17^ ions/cm^2^, a new phase Cr_2_Ti was detected in the samples with the detection accuracy limitation of XRD. The peak intensity of Cr_2_Ti phase increases gradually as the implantation dose further increased. That means that Ti ion penetration on the surface of 316L stainless steel was successfully achieved in this work and that the implanted Ti ion mainly existed in the form of Cr_2_Ti. Moreover, the improvement in implantation dose appeared to promote the formation of new phases. This phenomenon was attributed to thermodynamic driving forces, which were induced by cascade collisions during Ti ion implantation [22]. The diffraction peak intensity decreased when the specimens were implanted at 1.0 × 10^18^ ions/cm^2^, which was attributed to the formation of an amorphous layer at the surface of the sample. The thermal effect and radiation effect induced by Ti ion implantation were gradually enhanced as the implantation dose increased, which could improve the amount of new phases increased and could promote the grain size of new phases to grow. Therefore, the increase in diffraction peak intensity at 75 degree was observed when the implantation dose increased up to 1.7 × 10^18^ ions/cm^2^.

**Figure 2:**
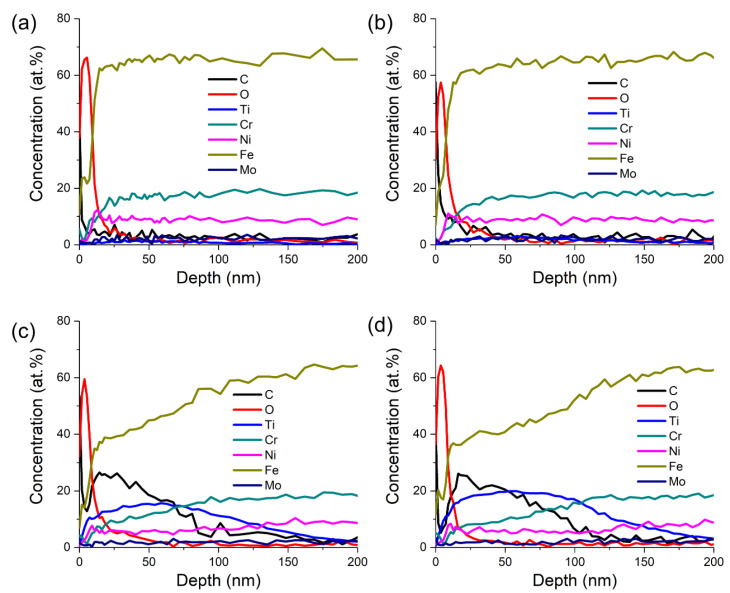
Elemental depth profiles of titanium-implanted samples obtained by sputtering Auger electron spectroscopy (AES) with different Ti ion-implanted doses: (**a**) 3.0 × 10^16^ ions/cm^2^, (**b**) 1.0 × 10^17^ ions/cm^2^, (**c**) 1.0 × 10^18^ ions/cm^2^, and (**d**) 1.7 × 10^18^ ions/cm^2^.

The depth profiles of seven elements (C, O, Ti, Cr, Ni, Fe, and Mo) as a function of depth for the samples with four different Ti ion implantation doses are shown in Figure 2. The increased concentration of the O element was observed at the surface for all the samples, which indicated that there was an oxidation layer formed on the surface during Ti ion implantation. The thickness of the oxidation layers was less than 50 nm, and the oxygen content is the highest at a depth of about 10 nm. When the Ti ion-implanted dose was less than 1.0 × 10^17^ ions/cm^2^, the concentrations of each element did not change significantly with depth, indicating that the implanted amount of Ti was very small at this time, which was consistent with the detection results of XRD. When the Ti ion-implanted dose was higher than 1.0 × 10^18^ ions/cm^2^, the concentrations of elements Ti and C increased significantly with the decrease in Fe concentration. With the increases in Ti ion implantation dose from 3.0 × 10^16^ to 1.7 × 10^18^ ions/cm^2^, the concentration of Ti increased from 1.6 to 19.9 at.%. The maximum Ti concentration was located at 20–100 nm below the surface.

**Figure 3:**
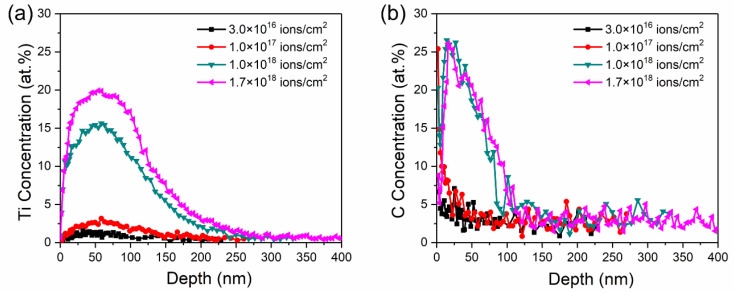
Elemental depth profiles of Ti (**a**) and C (**b**) of the samples with different Ti ion-implanted doses obtained by sputtering AES.

Figure 3 shows the Ti and C profiles with the depth of the samples at different Ti ion-implanted doses. The Ti profiles evolved from Gaussian-like profiles at low dose (≤1.0 × 10^17^ ions/cm^2^) to near sputter-limited profiles at higher dose (≥1.0 × 10^18^ ions/cm^2^). The penetration depth of Ti increased from 150 nm to 300 nm as the implanted dose increased. The increase in Ti ion implantation depth was mainly attributed to beam heating, which could help Ti atoms preferentially transport into the substrate through thermal diffusion and defect flux [23]. However, the implanted depth almost did not improve when the implanted dose was more than 1.0 × 10^18^ ions/cm^2^. This may be attributed to the dynamic balance between the injection atoms and sputtering off atoms.

Furthermore, a large concentration of C is detected during Ti ion implantation, as shown in Figure 3b. It was reported that C atom were bonded preferentially to Ti atom to form TiC at the surface of samples implanted with Ti ion in a previous study [24]. Therefore, it can be inferred that the increase in C concentration is caused by the diffusion of TiC formed on the surface. The profile of C concentration is similar to the diffusion curve, i.e., a high C concentration at or near the surface and a rapid decrease into the bulk. The retained concentration of C increased with the Ti ion implanted dose and basically remained unchanged when the dose was more than 1.0 × 10^18^ ions/cm^2^. It is supposed that the TiC molecules were formed by chemisorption between penetration Ti ions and carbonaceous gas molecules [25]. Therefore, the depth profiles of C concentration were changed with retained concentrations of Ti. Carbon had a beneficial effect on the improvement in tribological properties and corrosion behavior of implanted specimens, which could promote formation of the Fe–Ti–C ternary phase at the subsurface with the implanted Ti at high fluence [21]. The retained carbon could also stabilize the amorphous state in the Fe-Ti–C alloy [25]. Moreover, Ti–C dual implantation could improve the wear resistance and corrosion resistance of samples for the formation of new phases [26].

### 3.2. Surface Morphology

**Figure 4:**
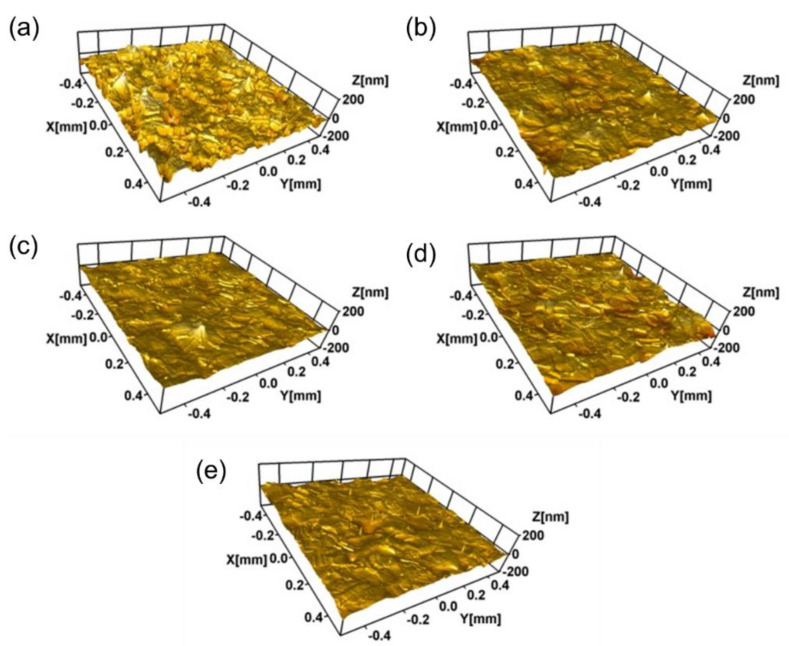
Nano 3D image (optical mode) of 316L stainless steel samples with different Ti ion implantation doses: (**a**) un-implanted; (**b**) 3.0 × 10^16^ ions/cm^2^; (**c**) 1.0 × 10^17^ ions/cm^2^; (**d**) 1.0 × 10^18^ ions/cm^2^, and (**e**) 1.7 × 10^18^ ions/cm^2^.

**Figure 5:**
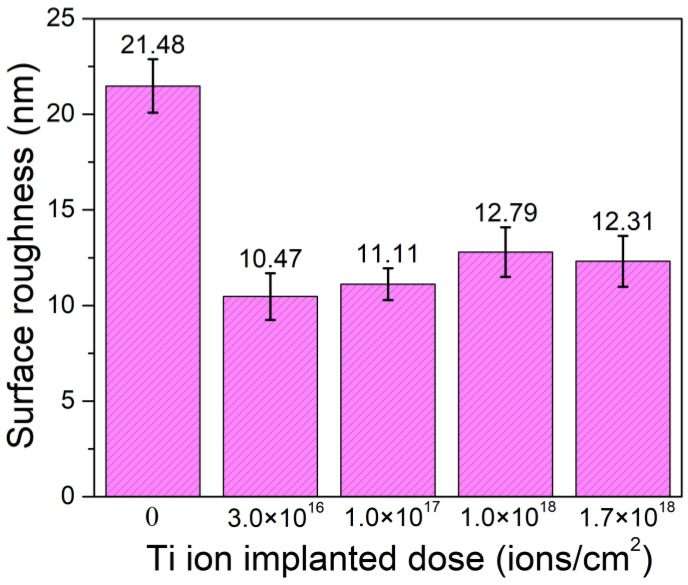
Surface roughness of the samples with different Ti ion-implanted doses.

Figure 4 shows the nano 3D images of the samples with different Ti ion-implanted does. It can be seen that the surfaces of all the samples were smooth and without obvious defect pits and particle adhesion. Furthermore, all the samples implanted with Ti ions presented smoother surfaces than that of the un-implanted samples. This is mainly due to sputter cleaning of the sample surface during the ion implantation process. Figure 5 shows the surface roughness of the samples with different doses of Ti ion implantation. It can be seen that the surface roughness of the samples with Ti ion implantation was much lower than that of the un-implanted sample. With the increase in Ti ion-implanted doses, the surface roughness of the samples slightly increased from 10.47 to 12.79 nm. When the implanted dose is small, less content from the elements penetrated into the substrate. At this time, ion sputtering mainly caused surface cleaning, resulting in a decrease in surface roughness. However, when the implanted dose was large, more content from the elements penetrated into the substrate, resulting in deformation of the surface and an increase in surface roughness.

### 3.3. Mechanical Property

**Figure 6:**
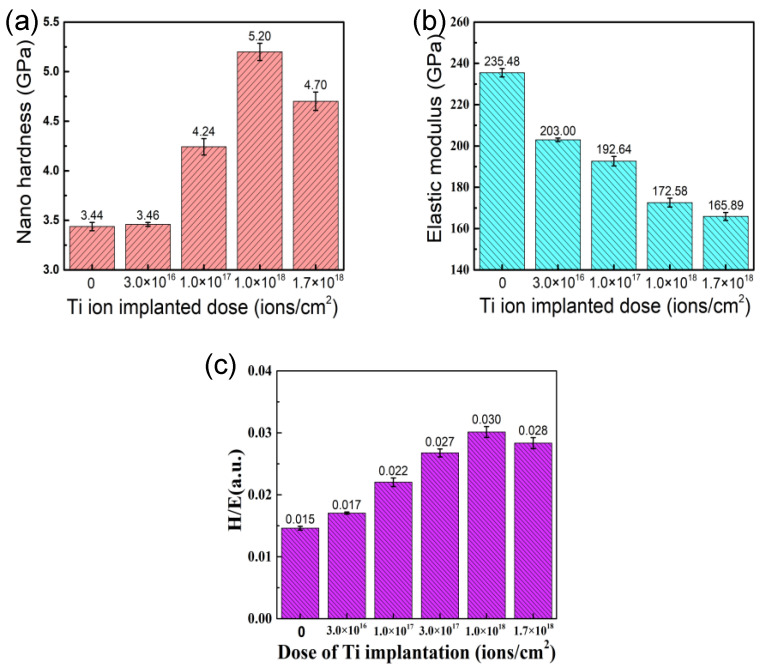
Mechanical properties of 316L stainless steel samples with different Ti ion implantation doses: (**a**) the surface hardness, (**b**) the surface elastic modulus, and (**c**) the ratio of hardness to elastic modulus (*H*/*E*).

Figure 6 shows nano-hardness, elastic modulus, and the calculated ratio of hardness and elastic modulus (*H/E*) of the samples with different doses of Ti ion implantation. The nano-hardness of the samples implanted with Ti ions is higher than that of the un-implanted sample. With the implantation dose increase, the nano-hardness of the samples first increased and then decreased, reaching a maximum value of 5.21 GPa at the dose 1.0 × 10^18^ ions/cm^2^. The elastic modulus of the samples gradually decreased from 235 to 166 GPa with the increase in implantation dose. Although hardness has always been considered the main material property defining wear resistance, it has been shown by a number of authors that the value of *H/E* may be more suitable in evaluating the wear resistance of materials than the hardness alone [27,28]. The *H/E* of the samples with different doses of Ti ion implantation were calculated in this work. With the implantation dose increase, the *H/E* increased at first and then decreased, reaching a maximum value of 0.03 at the dose 1.0 × 10^18^ ions/cm^2^, which is consistent with the changing trend of nano-hardness.

As is known, a large number of defects and solid solution phases were formed at the surface of samples implanted by Ti ion, which could promote surface hardening through defect hardening and solution hardening. Furthermore, the strengthening effect resulting from solution hardening and defect concentration improved with the increase in implantation dose [29]. When the implantation dose rose to a certain dose, it led to precipitation. A new stable intermetallic compound (Cr_2_Ti) was formed with the Ti ion implantation, which is in agreement with the XRD patterns. As shown by the XRD patterns and AES results, the resulting compounds (Cr_2_Ti and TiC) disperse in the implanted layer as fine precipitates at the grain boundary, which could hinder the movement of dislocations and realizes the enhancement of the sample surface. Although an amorphous layer was formed at the surface of the samples, the nano-hardness of samples still increased when the Ti implantation was raised up to 1.0 × 10^18^ ions/cm^2^, which contributes to the hardness of the sublayer-increased result from the long-effect induced by implantation [30]. It has been suggested that there is a critical dimension for precipitates. Corresponding to the given hardening mechanism, the effect of precipitate hardening is weakened when the dimension of secondary phases exceeded the critical value [31]. However, the formation of an amorphous phase at the surface was well expected with the decrease in elastic modulus at high implantation doses.

### 3.4. Friction and Wear

**Figure 7:**
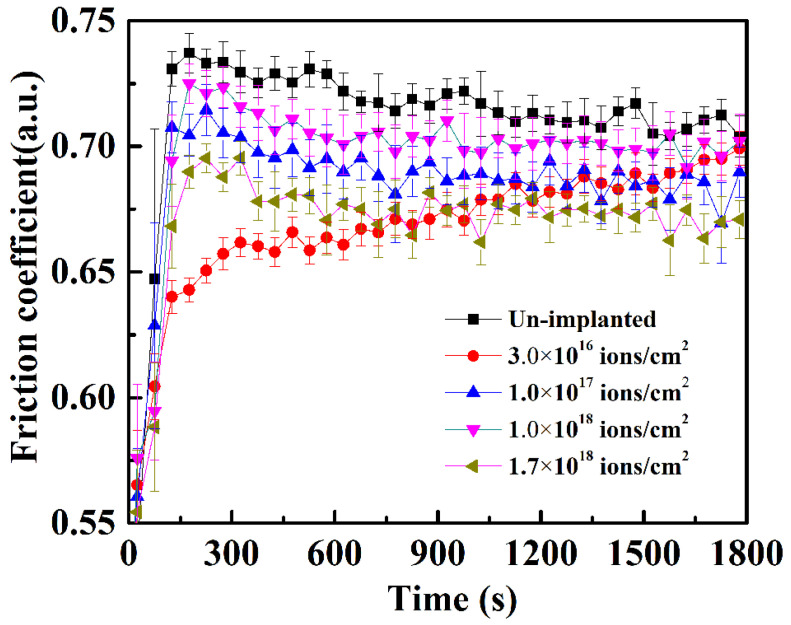
Friction coefficient of 316L stainless steel samples with different Ti ion implantation doses.

The friction coefficient of the samples before and after Ti ion implantation is illustrated in Figure 7. It can be seen that the friction coefficient of the samples with Ti ion implantation is lower than that of the un-implanted sample, which is owing to the lower surface roughness after Ti ion implantation. The friction coefficient at a dose of 3.0 × 10^16^ ions/cm^2^ reduces at first, benefiting from the implantation; then, it increased gradually during sliding due to the removal of the Ti ion-affected layer. The slight increase in the fiction coefficient at the dose 1.0 × 10^18^ ions/cm^2^ was caused by the increase in the surface roughness, as shown in Figure 2. The decrease in the friction coefficient, observed at the implanted dose 1.7 × 10^18^ ions/cm^2^, may be attributed to the formation of the Fe–Ti–C ternary amorphous phase, as reported by Singer and coworkers [21].

**Figure 8:**
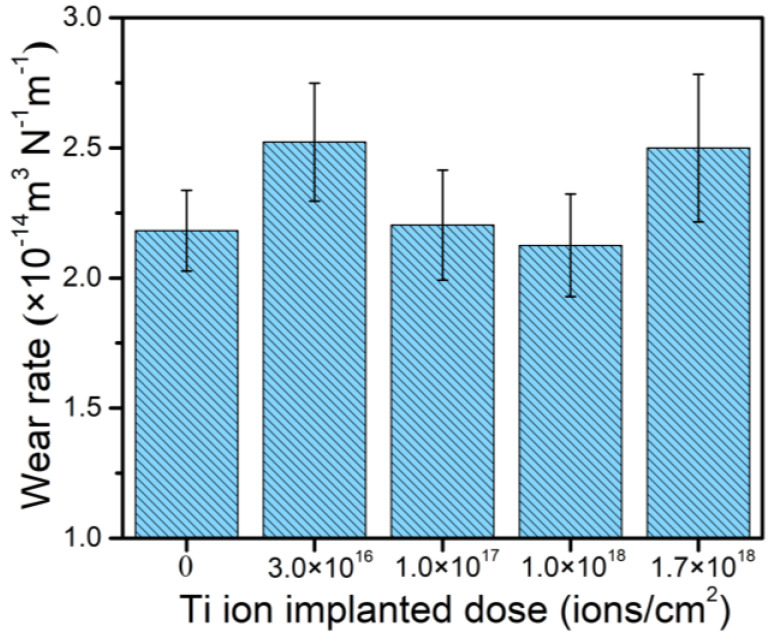
Wear rate of 316L stainless steel samples with different Ti ion implantation doses.

According to previous research [27,28], an increase in hardness and *H*/*E* is beneficial to the improvement of wear resistance and *H/E* has an important effect on the tribological properties of modified layers. Materials with higher *H*/*E* are expected to have a lower friction and higher wear resistance. Hence, a lot of researchers use it to represent the plastic deformation resistance of modified layers. Figure 8 shows the calculated wear rate of the samples with different doses of Ti ion implantation. The wear rate decreases with the implantation dose increasing up to 1.0 × 10^18^ ions/cm^2^ and then increases when the dose is raised up to 1.7 × 10^18^ ions/cm^2^. The trend in wear resistance coincides with the hardness and *H/E* of the samples very well, as shown in Figure 6. The improvement in wear resistance may be attributed to surface strengthening, resulting from the creation of defects, the formation of new precipitate (Cr_2_Ti), and solid solutions during Ti ion implantation. A decrease in wear resistance was observed for the sample with Ti ion implantation at the dose 1.7 × 10^18^ ions/cm^2^, which may be associated with coarsening of the formed precipitates. As reported by Madakson [32], the mechanical properties of modified samples would deteriorate when the size and shape of the precipitates exceed a critical value and special shape.

**Figure 9:**
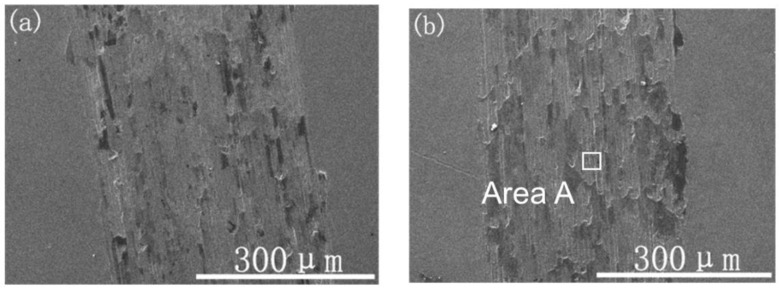

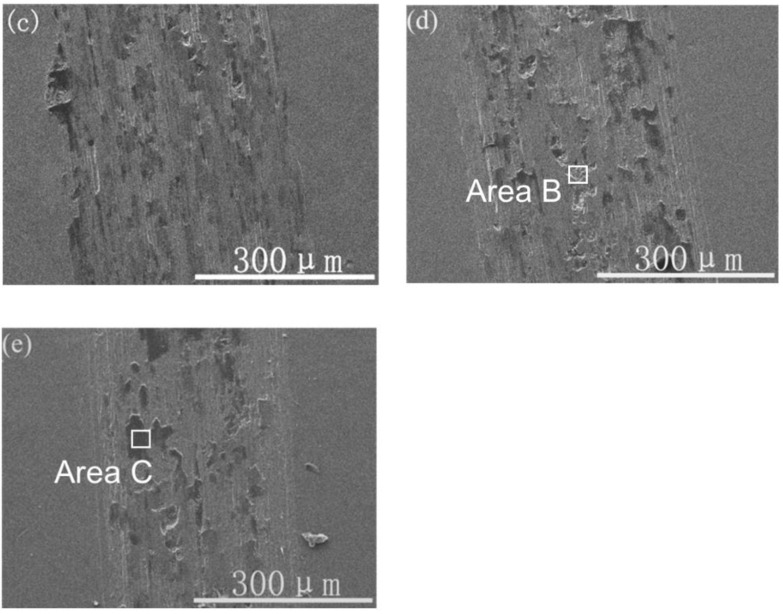
SEM images of the wear track topography of the samples with different Ti ion-implanted doses: (**a**) un-implanted, (**b**) 3.0 × 10^16^ ions/cm^2^, (**c**) 1.0 × 10^17^ ions/cm^2^, (**d**) 1.0 × 10^18^ ions/cm^2^, and (**e**) 1.7 × 10^18^ ions/cm^2^.

Figure 9 presents the typical SEM images of the wear tracks of the samples with different Ti ion-implanted doses. Wear debris and peeling pits were observed in the wear track of the Ti ion-implanted samples. According to EDS analysis of the black areas (area C), as shown in Table 1, the metal surfaces were serious oxidized in the atmosphere by frictional heating during sliding. The element Si detected in area C migrated from the grinding ball material. The Ti was not detected by EDS in area B, which shows that the microcrack propagation caused by adhesive wear occurs on the lower surface of the ion implantation layer. According to the above analysis, the content of black areas (area C) can be used to characterize the plastic deformation resistance and oxidation resistance of the material surface. As shown in Figure 9, the content of the black areas reduced with a dose increase up to 1.0 × 10^18^ ions/cm^2^, it was slightly increased when Ti was implanted at a dose of 1.7 × 10^18^ ions/cm^2^. Therefore, the antioxidant capacity of samples increased as the dose increased up to 1.0 × 10^18^ ions/cm^2^, for which the trend is in very good accordance with the results of *H/E*. As shown in a previous study [21], Ti was a chemically active element that easily forms a TiO_2_ layer. The TiO_2_ layer likely forms a sandwich structure between a thin outer Fe_2_O_3_ layer and the metallic substrate, which may prevent the formation of Fe-oxide during wear, resulting in a reduction in wear.

Moreover, plenty of microgrooves and abrasive wear were observed on the wear tracks in the Ti ion-implanted samples. These microgrooves were probably caused by the precipitation of hard particles (Cr_2_Ti), which scratched the surface of the samples during sliding. The wear morphology suggested that the wear mechanisms of Ti ion-implanted samples are mainly adhesive and oxidative wear with slight abrasive wear.

## 4. Conclusions

This research provided the chemical composition, surface morphology, surface hardness, and wear properties of 316L stainless steel implanted with different doses of Ti ions. A new phase (Cr_2_Ti) was detected, and the concentrations of Ti and C increased obviously when the dose exceeded 1.0 × 10^17^ ions/cm^2^. The surface roughness can be significantly reduced after Ti ion implantation. The nano-hardness increased from 3.44 to 5.21 GPa as the Ti ion implanted dose increased up to 1.0 × 10^18^ ions/cm^2^. The friction coefficient decreased from 0.78 for un-implanted samples to 0.68 for samples at doses of 1.7 × 10^18^ ions/cm^2^. The wear rate was slightly improved when the sample implanted Ti ion at the dose of 1.0 × 10^18^ ions/cm^2^. Adhesive wear and oxidation wear are the main wear mechanisms, and a slightly abrasive wear is observed during sliding. Oxidation wear improved significantly as the implantation dose increased.

## Figures and Tables

**Table 1:** EDS analysis result of wear track topography.

Element	O	Si	Ti	Cr	Mn	Fe	Ni	Mo
A (at.%)	14.55	1.07	0.73	14.54	0.46	58.16	9.75	0.73
B (at.%)	11.37	1.15	0	17.03	0.26	60.43	8.67	1.08
C (at.%)	40.71	5.19	0.32	10.23	0.87	36.62	5.03	1.02

## Data Availability

Not applicable for the studies not reporting any data.
